# Aberrant plasma IL-7 and soluble IL-7 receptor levels indicate impaired T-cell response to IL-7 in human tuberculosis

**DOI:** 10.1371/journal.ppat.1006425

**Published:** 2017-06-05

**Authors:** Christian Lundtoft, Anthony Afum-Adjei Awuah, Jens Rimpler, Kirstin Harling, Norman Nausch, Malte Kohns, Ernest Adankwah, Franziska Lang, Laura Olbrich, Ertan Mayatepek, Ellis Owusu-Dabo, Marc Jacobsen

**Affiliations:** 1 Department of General Pediatrics, Neonatology, and Pediatric Cardiology, University Children’s Hospital, Medical Faculty, Duesseldorf, Germany; 2 Kumasi Centre for collaborative Research in Tropical Medicine (KCCR), Kumasi, Ghana; 3 School of Medical Sciences, Kwame Nkrumah University of Science and Technology (KNUST), Kumasi, Ghana; Portland VA Medical Center, Oregon Health and Science University, UNITED STATES

## Abstract

T-cell proliferation and generation of protective memory during chronic infections depend on Interleukin-7 (IL-7) availability and receptivity. Regulation of IL-7 receptor (IL-7R) expression and signalling are key for IL-7-modulated T-cell functions. Aberrant expression of soluble (s) and membrane-associated (m) IL-7R molecules is associated with development of autoimmunity and immune failure in acquired immune deficiency syndrome (AIDS) patients. Here we investigated the role of IL-7/IL-7R on T-cell immunity in human tuberculosis. We performed two independent case-control studies comparing tuberculosis patients and healthy contacts. This was combined with follow-up examinations for a subgroup of tuberculosis patients under therapy and recovery. Blood plasma and T cells were characterised for IL-7/sIL-7R and mIL-7R expression, respectively. IL-7-dependent T-cell functions were determined by analysing STAT5 phosphorylation, antigen-specific cytokine release and by analysing markers of T-cell exhaustion and inflammation. Tuberculosis patients had lower soluble IL-7R (p < 0.001) and higher IL-7 (p < 0.001) plasma concentrations as compared to healthy contacts. Both markers were largely independent and aberrant expression normalised during therapy and recovery. Furthermore, tuberculosis patients had lower levels of mIL-7R in T cells caused by post-transcriptional mechanisms. Functional *in vitro* tests indicated diminished IL-7-induced STAT5 phosphorylation and impaired IL-7-promoted cytokine release of *Mycobacterium tuberculosis*-specific CD4^+^ T cells from tuberculosis patients. Finally, we determined T-cell exhaustion markers PD-1 and SOCS3 and detected increased SOCS3 expression during therapy. Only moderate correlation of PD-1 and SOCS3 with IL-7 expression was observed. We conclude that diminished soluble IL-7R and increased IL-7 plasma concentrations, as well as decreased membrane-associated IL-7R expression in T cells, reflect impaired T-cell sensitivity to IL-7 in tuberculosis patients. These findings show similarities to pathognomonic features of impaired T-cell functions and immune failure described in AIDS patients.

## Author summary

IL-7 is important for the development and homeostasis of T cells and promotes antigen-specific T-cell responses. Aberrant expression of plasma IL-7 and soluble IL-7R are found in autoimmune diseases and chronic viral infections. In AIDS patients—especially those who fail to reconstitute T-cell numbers during therapy—impaired IL-7-promoted T-cell functions indicated T-cell exhaustion/senescence. In order to evaluate the potential impact of IL-7 on tuberculosis, we characterised various parameters involved in the IL-7-response of tuberculosis patients and healthy contacts. Despite IL-7 being available at higher plasma levels among tuberculosis patients, the T-cell response to IL-7 was impaired when compared to healthy contacts. Soluble IL-7R levels were aberrantly low in plasma during acute tuberculosis but did not account for impaired IL-7 usage. Chronic inflammation in tuberculosis patients—reflected by increased IL-6 plasma levels—did not account for dysfunctional T-cell responses and analysed T-cell exhaustion markers were only moderately correlated. Our findings demonstrate that availability of IL-7 alone is not sufficient to promote protective T-cell immunity against tuberculosis. We describe aberrant IL-7/soluble IL-7R expression and impaired IL-7-mediated T-cell functions in tuberculosis patients with similarities and differences to described IL-7 dysregulation seen in patients with AIDS.

## Introduction

T cells are crucial for protection against *Mycobacterium (M*.*) tuberculosis* infection but biomarkers that characterise T-cell failure and progression towards tuberculosis disease are not available [1]. CD4^+^ T cells are key to anti-mycobacterial immune protection [2] and CD4^+^ T-cell deficiency, e.g. of AIDS patients, results in increased susceptibility against tuberculosis [3–5]. There is growing evidence that impaired CD4^+^ T-cell functions play a role in tuberculosis [6]. Recent studies identified T-cell exhaustion as a feature of tuberculosis [7, 8]. T-cell exhaustion impairs immunity against chronic viral infections and harms memory T-cell potential [9]. IL-7 is central for generation of memory T cells and was shown to revert T-cell exhaustion in chronic viral infections [10]. Notably, IL-7 induced T-cell memory was hampered in the presence of persistent antigen and inflammation as seen for chronic viral infections [11]. In AIDS patients, failure of immune reconstitution is accompanied by a dysfunctional T-cell response that showed features of senescence and exhaustion [12–14]. Recently, persistent inflammation characterised e.g. by increased IL-6 serum concentrations from AIDS patients were found to correlate with T-cell exhaustion/senescence and impaired T-cell response to IL-7 [14, 15]. High IL-7 plasma levels as well as decreased membrane-associated (m)IL-7R expression of T cells were found in AIDS patients with immune failure [16, 17]. Concomitantly impaired T-cell response to IL-7 was detected in immune failure patients [13–15, 18–20].

The regulation of IL-7R expression is central for control of IL-7-mediated effects on T cells [21]. On IL-7 binding, the mIL-7R assembles as a heterodimer (comprising the IL-7Rα (CD127) and the common γ-chain (CD132)) and induces signalling cascades mainly via the Jak/STAT pathway. Jak1 and Jak3 are involved in IL-7R signalling, and STAT5 gets phosphorylated and initiates multiple transcription events [22]. As part of IL-7 signalling, the mIL-7R is rapidly internalised, becomes partly degraded or recycles to the cell surface [23]. Regulation of IL-7R expression is also controlled on the transcriptional level and IL-7 and other cytokines were shown to suppress IL-7R mRNA expression [24]. Alternative splicing of the *IL7RA* gene generates a soluble IL-7R (sIL-7R) variant [25]. The sIL-7R variant binds IL-7 although with lower affinity as compared to the mIL-7R heterodimer and is present in blood plasma at high molar excess relative to IL-7 [26]. The exact role of the sIL-7R for IL-7 metabolism remains elusive. Competitive inhibition of IL-7 uptake as well as IL-7 reservoir functions have been described [26–28]. Differential sIL-7R plasma concentrations are found in immune pathologies, e.g. autoimmune diseases [26, 29, 30] and AIDS [28, 31]. In addition, a functional *IL7RA* polymorphism (rs6897932) that interferes with IL-7R alternative splicing and thereby leads to reduced sIL-7R levels in plasma was found to be associated with autoimmune diseases [32, 33] and to affect immune reconstitution in AIDS patients [34–36].

Initial results indicating a role of IL-7 during T-cell immunity against tuberculosis were derived from animal models. Increased IL-7 and soluble IL-7R expression in pulmonary tissue of primates with tuberculosis was found, indicating a possible role of IL-7 metabolism in tuberculosis pathogenesis [37, 38]. Furthermore IL-7 was shown to promote survival and to improve BCG vaccination efficacy in *M*. *tuberculosis*-infected mice [39, 40]. However, a comprehensive understanding of the possible role of IL-7 or IL-7R functions in human tuberculosis has not yet been developed.

This present study aimed to elucidate a possible role of IL-7 modulated T-cell responses in human tuberculosis. We determined sIL-7R and IL-7 plasma concentrations and mIL-7R expression of T cells from tuberculosis patients—before, during, and after chemotherapy—and compared these to healthy contacts. Since results resembled pattern seen in AIDS patients with impaired T-cell response to IL-7, we then performed functional T-cell assays in a second set of tuberculosis patients and healthy contacts to determine IL-7-mediated signalling and promoted cytokine release on *M*. *tuberculosis*-specific T-cell activation. Finally, mRNA expression of exhaustion markers was compared in CD4^+^ T cells between the cohorts to evaluate a possible causative role of T-cell exhaustion for impaired IL-7 response in tuberculosis.

## Results

### Decreased sIL-7R plasma concentrations in acute tuberculosis patients

**Fig 1:**
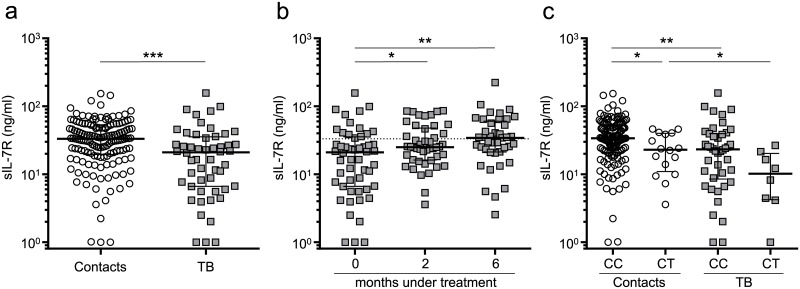
Plasma sIL-7R level in healthy contacts and tuberculosis during chemotherapy. **(a)** Concentrations of sIL-7R in plasma from tuberculosis patients (n = 52) and healthy contacts (n = 149) determined by cytometric bead array. **(b)** sIL-7R plasma concentration prior to (0 months, n = 52), during (2 months, n = 46) and after (6 months, n = 41) treatment of tuberculosis patients. Median sIL-7R plasma concentration of healthy contacts is indicated with a dotted line. **(c)** Plasma sIL-7R levels stratified for the *IL7RA* exon 6 single nucleotide polymorphism rs6897932C>T for healthy contacts (n = 142) and tuberculosis patients (n = 50). Median and interquartile range is depicted, and each symbol indicates mean values of duplicates from each individual donor. Exact Mann-Whitney U test was used for comparison of groups, while paired data was evaluated by Wilcoxon Signed-Rank test. Nominal p-values are indicated as: * p < 0.05, ** p < 0.01, *** p < 0.001.

Aberrant sIL-7R plasma levels indicate pathologic T-cell immunity in autoimmune, inflammatory, and chronic viral diseases. Hence, we determined sIL-7R plasma concentrations in individuals infected with *M*. *tuberculosis*. Patients with active tuberculosis (n = 57) and healthy contacts (n = 151) were included. Tuberculosis patients had significantly lower sIL-7R concentrations as compared to healthy contacts (p < 0.001) (Fig 1a). Since study groups differed in gender distributions (tuberculosis: 30% females; contacts: 56% females; Table 1), we compared sIL-7R between male and female subgroups. Female patients with tuberculosis showed moderately lower sIL-7R concentrations as compared to male patients, whereas no differences were detected for healthy contacts (S1 Fig). Therefore, differences in plasma sIL-7R were not due to gender differences. Next we determined the influence of anti-tuberculosis therapy and recovery on plasma sIL-7R in tuberculosis patients (i.e. 2 months and 6 months after therapy onset). Analyses revealed significantly increased sIL-7R plasma levels after 2 months (p = 0.03) and after recovery (p = 0.009) (Fig 1b). sIL-7R plasma concentrations of recovered tuberculosis patients were comparable to healthy contacts (Fig 1b). To determine if changes in sIL-7R under therapy were dependent on sIL-7R concentrations prior to treatment, we compared initial sIL-7R concentrations with changes of sIL-7R expression between 0 and 6 months. Absolute differences and ratios were calculated. Absolute differences (month 6 –month 0) showed only moderate negative correlation with initial sIL-7R levels (rho = -0.26; p = 0.13) (S2a Fig), but changes of ratios (month 6 / month 0) were strongly associated with sIL-7R levels prior to treatment (rho = -0.61, p < 0.001) (S2a Fig). Therefore, especially tuberculosis patients with low sIL-7R concentrations prior to treatment showed increased sIL-7R levels after recovery and a relative gain of sIL-7R plasma concentration was detected.

**Table 1:** Patient characteristics.

Cohort 1	Healthy Contacts	TB
Number of participants	151	57
Age (y)	31 [18–68]	33 [18–71]
Gender		
Female	84 (56%)	17 (30%)
Male	67 (44%)	40 (70%)
BCG vaccination		
Yes	90 (60%)	28 (49%)
No	50 (33%)	28 (49%)
No information	11 (7%)	1 (2%)
**Cohort 2**		
Number of participants	24	22
Age (y)	41 [21–65]	39 [15–72]
Gender		
Female	14 (58%)	15 (68%)
Male	10 (42%)	7 (32%)

Median [range] or number (proportion) is shown.

### The IL7RA functional polymorphism rs6897932 contributed to differential sIL-7R plasma levels

A functional single nucleotide polymorphism (SNP, rs6897932C>T) in exon 6 of the *IL7RA* gene interferes with splicing and impairs sIL-7R expression [32]. Therefore, we determined the rs6897932 minor T allele (rs6897932T) frequency in tuberculosis patients and healthy contacts. Tuberculosis patients had a marginally higher MAF proportion (7.3%) as compared to healthy contacts (5.6%). No homozygous rs6897932T/T carriers were identified in the study groups. As expected, lower levels of plasma sIL-7R were detected for rs6897932C/T healthy contacts as compared to rs6897932C/C wild type healthy contacts (p = 0.02), and the same tendency was seen for the tuberculosis patients (p = 0.06) (Fig 1c). However, stratification for SNP genotypes confirmed lower plasma sIL-7R among tuberculosis patients when compared to healthy contacts (p < 0.001). We concluded that increased frequencies of IL-7R rs6897932T alleles in tuberculosis patients contributed to differential sIL-7R levels but did not account for lower sIL-7R plasma concentrations of tuberculosis patients.

### Increased IL-7 plasma concentrations in tuberculosis patients but no correlation with sIL-7R

**Fig 2:**
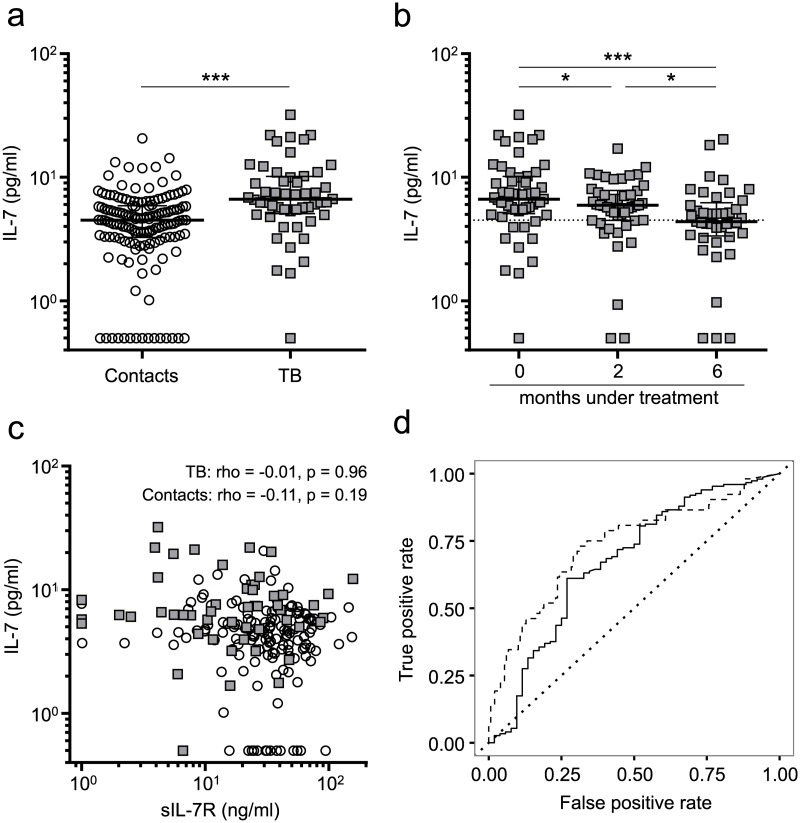
Plasma IL-7 concentration of tuberculosis patients and healthy contacts. **(a)** Comparison of IL-7 plasma concentrations between tuberculosis patients (n = 52) and healthy contacts (n = 148). **(b)** Plasma IL-7 concentrations prior to (0 months, n = 52), during (2 months, n = 46), and after (6 months, n = 41) treatment of tuberculosis patients. Median IL-7 plasma level of healthy contacts is represented with a dotted line. **(c)** Spearman correlation between plasma IL-7 and sIL-7R for tuberculosis patients prior to treatment (squares), and healthy contacts (circles). **(d)** Receiver operating characteristic (ROC) curve indicates sensitivity and specificity of plasma sIL-7R (solid line) and plasma IL-7 (dashed line) to discriminate between patients with tuberculosis and healthy contacts. The line of no discrimination is indicated as a dotted line. Median and interquartile range is depicted, and each symbol indicates mean values of duplicates from each individual donor. Exact Mann-Whitney U test was used for comparison of groups, whereas paired data was evaluated by Wilcoxon Signed-Rank test. Nominal p-values are indicated as: * p < 0.05, *** p < 0.001.

We hypothesised that differential sIL-7R plasma levels would affect IL-7 consumption. Consequently we next determined IL-7 plasma concentrations in tuberculosis patients and healthy contacts. Tuberculosis patients showed significantly increased IL-7 concentrations prior to therapy as compared to healthy contacts (p < 0.001) (Fig 2a). IL-7 concentrations decreased under therapy and recovery (0 vs. 6 months, p < 0.001) and reached levels comparable to healthy contacts (Fig 2b). Higher initial IL-7 levels were associated with stronger decrease rates until month 6 (rho = -0.58, p < 0.001; S2b Fig). Notably, and in contrast to sIL-7R results, also absolute differences between month 0 and 6 correlated strongly with IL-7 levels prior to therapy (rho = -0.79, p < 0.001; S2b Fig). This indicated different mechanisms involved in IL-7 and sIL-7R regulation during tuberculosis. In accordance, no dependency was detected between IL-7 and sIL-7R plasma concentrations for tuberculosis patients or healthy contacts (Fig 2c).

These results suggested that IL-7 and sIL-7R could be useful as biomarkers for diagnosis of tuberculosis patients. Comparison of tuberculosis patients and healthy contacts revealed moderate discrimination capacity for both sIL-7R (AUC = 0.67) and IL-7 (AUC = 0.73) using Receiver Operating Characteristic (ROC) analysis (Fig 2d). Independency of IL-7 and sIL-7R plasma levels (Fig 2c) prompted us to calculate the combined efficacy of both markers using Random Forest analysis (for details see Methods section). Correct prediction of tuberculosis patients and healthy contacts was achieved for 73% of all donors, and IL-7 was about two times more influential on prediction than sIL-7R. These results indicated that IL-7 and sIL-7R plasma concentrations were largely independent and may contribute to tuberculosis diagnosis.

### Increased proportions of mIL-7R_low_ CD4^+^ and CD8^+^ T cells in tuberculosis patients

**Fig 3:**
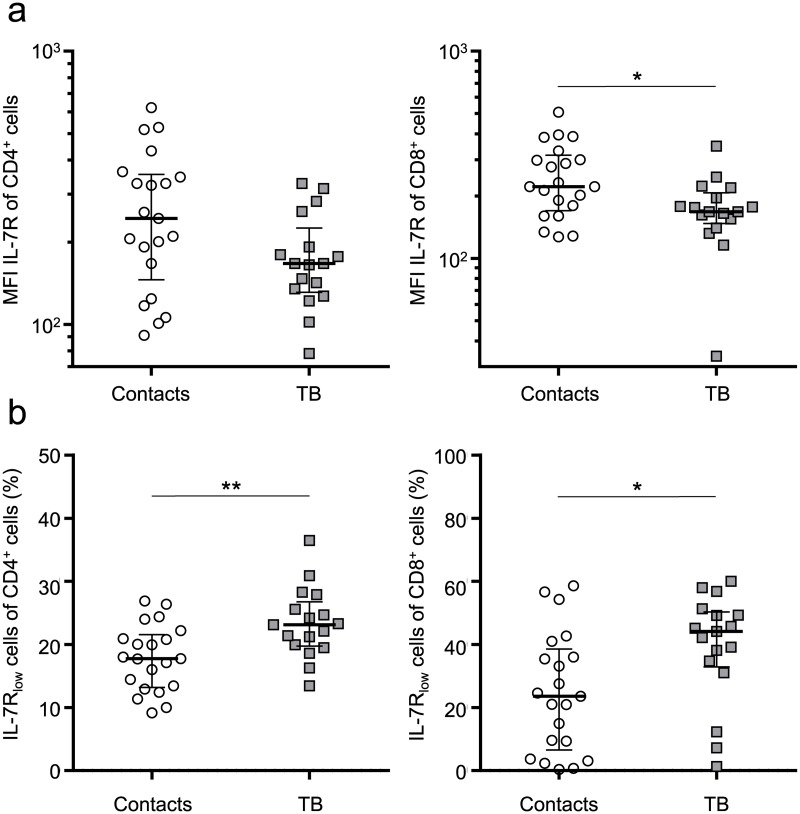
Membrane-associated IL-7R expression on CD4^+^ and CD8^+^ T cells. Membrane-associated IL-7R expression of CD4^+^ (left graphs) and CD8^+^ (right graphs) T cells from tuberculosis patients (n = 17) and healthy contacts (n = 21) analysed by flow cytometry. **(a)** Mean fluorescence intensity (MFI) analyses of IL-7R. **(b)** Proportions of IL-7R_low_-expressing CD4^+^ and CD8^+^ T cells from tuberculosis patients and healthy contacts. Median and interquartile range is depicted, and each symbol indicates mean values of duplicates from each individual donor. Exact Mann-Whitney U test used for comparison of groups. Nominal p-values are indicated as * p < 0.05, ** p < 0.01.

Increased IL-7 plasma concentrations are likely caused by decreased T-cell consumption of IL-7. Low T-cell numbers or impaired T-cell receptivity of IL-7 may account for this. Hence we compared mIL-7R protein expression for subgroups of tuberculosis patients and healthy contacts by flow cytometry. We detected lower mean mIL-7R expression for CD8^+^ T cells (p = 0.02) and a tendency for CD4^+^ T cells (p = 0.05) (Fig 3a). Analysis of mIL-7R on T-cell subpopulations revealed increased proportions of mIL-7R_low_ CD4^+^ (p = 0.006) and CD8^+^ T cells (p = 0.02) from tuberculosis patients as compared to healthy contacts (Fig 3b). To confirm these observations, we performed mIL-7R analysis in a second independent cohort study including additionally recruited tuberculosis patients (n = 22) and healthy contacts (n = 24). Due to restriction in the number of flow cytometry parameters, CD4^+^ and CD4^-^ T cells were analysed for mIL-7R protein expression. Tuberculosis patients showed significantly decreased mIL-7R expression for both CD4^+^ (p = 0.01) and CD4^-^ (p = 0.006) T cells (S4 Fig). This confirmed initial results and led us to the conclusion that impaired mIL-7R expression of T cells resulted in increased proportions of mIL-7R_low_ CD4^+^ and CD8^+^ T cells in tuberculosis patients.

Differential mIL-7R expression may be affected by plasma IL-7 and sIL-7R levels. We determined correlation between these parameters to identify possible interactions. A tendency of positive correlation between mIL-7R expression and sIL-7R plasma (rho = 0.42, p = 0.06) was found only in the group of contacts, whereas mIL-7R and IL-7 showed a marginal negative correlation (rho = -0.38, p = 0.10) (S1 Table) in this study group. No correlation between any parameters was found for tuberculosis patients (S1 Table).

### Similar IL-7R isoform mRNA expression of CD4^+^ T cells from tuberculosis patients and healthy contacts

**Fig 4:**
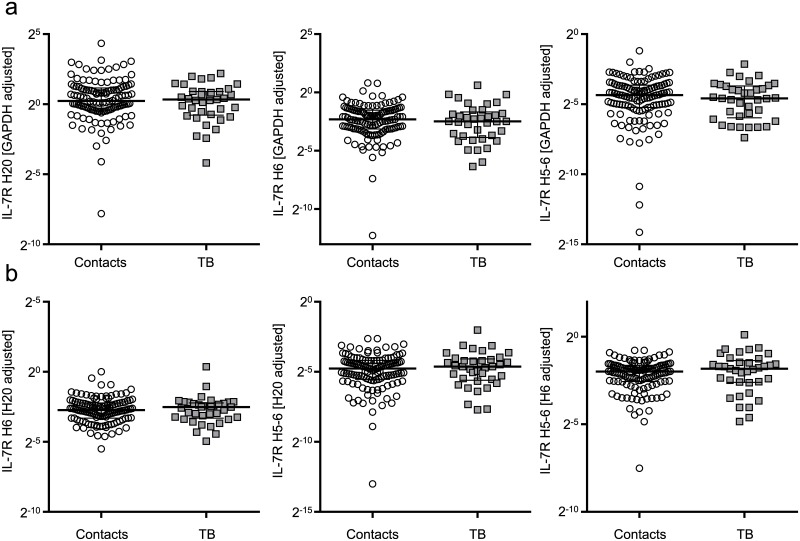
mRNA expression of IL-7R variants of CD4^+^ T cells from tuberculosis patients and healthy contacts. The expression of full length IL-7R (H20), and IL-7R lacking exon 6 (H6) or exon 5–6 (H5-6) was evaluated using mRNA isolated from CD4^+^ T cells. **(a)** Glyceraldehyde 3-phosphate dehydrogenase (GAPDH), or **(b)** IL-7R variants were applied as references. Cycle threshold differences (2^-ΔCt^) are shown for tuberculosis patients (n = 37) and healthy contacts (n = 120). Median and interquartile range is depicted, and each symbol indicates mean values of duplicates from each individual donor. Exact Mann-Whitney U test used for comparison of groups.

High IL-7 plasma levels and low mIL-7R expression of T cells have previously been described for HIV/AIDS patients [16, 17, 41, 42]. In AIDS patients these differences are accompanied with mIL-7R regulatory dysfunctions [43]. Therefore we questioned whether aberrant expression of IL-7R variants in tuberculosis patients is caused by differential regulation on the transcriptional or post-transcriptional level. Hence, we analysed IL-7R mRNA transcripts of purified CD4^+^ T cells from tuberculosis patients and healthy contacts. Three IL-7R transcripts coding for the mIL-7R (all 8 exons included; H20) and a sIL-7R (H6 and H5-6; for details see Methods section [25]) were measured. None of the IL-7R variants were differentially expressed on the mRNA level of CD4^+^ T cells between tuberculosis patients and healthy contacts (Fig 4a). Also relative expression of sIL-7R vs. mIL-7R transcripts was similar between study groups (Fig 4b). These results indicated that differential IL-7R mRNA expression is not the cause for aberrant sIL-7R and mIL-7R expression in tuberculosis patients and render causative post-transcriptional mechanisms likely.

### Impaired IL-7-induced STAT5 phosphorylation and IL-7-promoted T-cell cytokine release in tuberculosis patients

**Fig 5:**
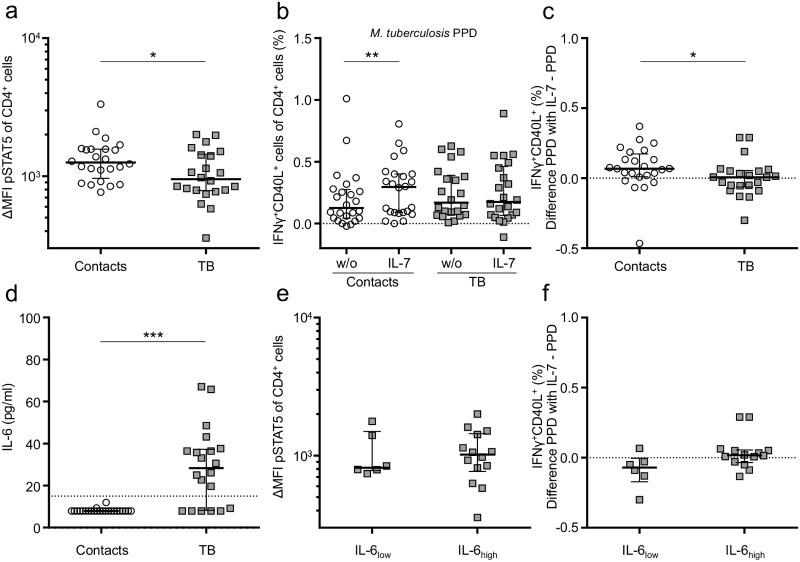
IL-7 response of CD4^+^ T cells from tuberculosis patients and healthy contacts. **(a)** IL-7-induced (10 ng/ml) STAT5 phosphorylation of CD4^+^ T cells from tuberculosis patients (n = 22) and healthy contacts (n = 24) measured by flow cytometry. The STAT5 phosphorylation level of non-stimulated cells has been subtracted of all values. **(b)** IFNγ/CD40L-expressing CD4^+^ T cells after PPD re-stimulation in the presence or absence of IL-7 detected by flow cytometry. Non-stimulated values with or without IL-7 have been subtracted. **(c)** Induction of IFNγ/CD40L-expressing CD4^+^ T cells by IL-7 and PPD stimulation. Absolute differences as compared to PPD alone are shown. **(d)** Plasma IL-6 levels of tuberculosis patients (n = 20) and healthy contacts (n = 24). An arbitrary threshold indicated by a dotted line was set to define tuberculosis patients with high (IL-6_high_) and low (IL-6_low_) concentrations of plasma IL-6. **(e)** STAT5 phosphorylation, and IL-7-induced PPD response **(f)** for tuberculosis patients with high or low plasma IL-6 level, as defined in (d). Median and interquartile range is depicted, and exact Mann-Whitney U test was applied for comparison of groups, whereas paired data was evaluated by Wilcoxon Signed-Rank test. Nominal p-values are indicated as * p < 0.05, ** p < 0.01, *** p < 0.001.

Impaired IL-7 signalling has been associated with diminished IL-7R_low_ expression of T cells from AIDS patients, but different mechanisms about the role of STAT5 were described [18, 44, 45]. To evaluate the effect of IL-7 signalling, we recruited a second cohort of tuberculosis patients (n = 22) or healthy contacts (n = 24) (Table 1). A lower surface level of mIL-7R on T cells from tuberculosis patients was confirmed in this cohort (S4 Fig). Next, we measured IL-7-induced STAT5 phosphorylation and detected decreased phosphorylated STAT5 in CD4^+^ T cells from tuberculosis patients as compared to healthy contacts (p = 0.04) (Fig 5a). Since IL-7 was shown to enhance specific T-cell cytokine release [46], we determined intracellular cytokines after *M*. *tuberculosis* antigen (PPD) *in vitro* stimulation in the presence or absence of IL-7. No differences were detected for PPD-specific T cells co-expressing IFNγ and CD40L when comparing tuberculosis patients and healthy contacts (Fig 5b). However, co-stimulation with IL-7 induced increased proportions IFNγ-producing T cells solely in the study group of healthy contacts (p = 0.003), but not in tuberculosis patients (p = 0.94) (Fig 5b). Next, IL-7-specific effects were quantified by calculating the difference of PPD induced T cells with or without IL-7 for each individual (Fig 5c). We found a significantly stronger effect of IL-7 on cytokine release in healthy contacts as compared to tuberculosis patients (p = 0.02). These results suggested impaired T-cell responses to IL-7 in patients with tuberculosis.

### Increased IL-6 plasma concentrations in tuberculosis patients were not associated with impaired T-cell responses to IL-7

Chronic inflammation and increased IL-6 serum concentrations were found in AIDS patients with impaired T-cell immunity to IL-7 [14, 15]. One study found a direct inhibitory effect of IL-6 on IL-7-mediated T-cell functions [15]. Since increased IL-6 plasma levels were described in tuberculosis previously [47], we measured plasma IL-6 levels and detected increased IL-6 concentrations in tuberculosis patients as compared to healthy contacts (p < 0.001) (Fig 5d). The distribution of IL-6 plasma concentrations indicated two subgroups of tuberculosis patients. Hence we set an arbitrary threshold (15 pg/ml) and compared IL-6_high_ and IL-6_low_ tuberculosis patients for IL-7-promoted T-cell responses. No significant differences in IL-7-induced STAT5 phosphorylation or IL-7 co-stimulated IFNγ/CD40L expression was found between the two IL-6_high_ and IL-6_low_ subgroups of tuberculosis patients (Fig 5e and 5f). Therefore differential IL-6 serum levels were not associated with impaired IL-7-promoted T-cell responses in tuberculosis patients.

### Exhaustion markers PD-1 and SOCS3 were not associated with IL-7-impaired T-cell response in tuberculosis patients

**Fig 6:**
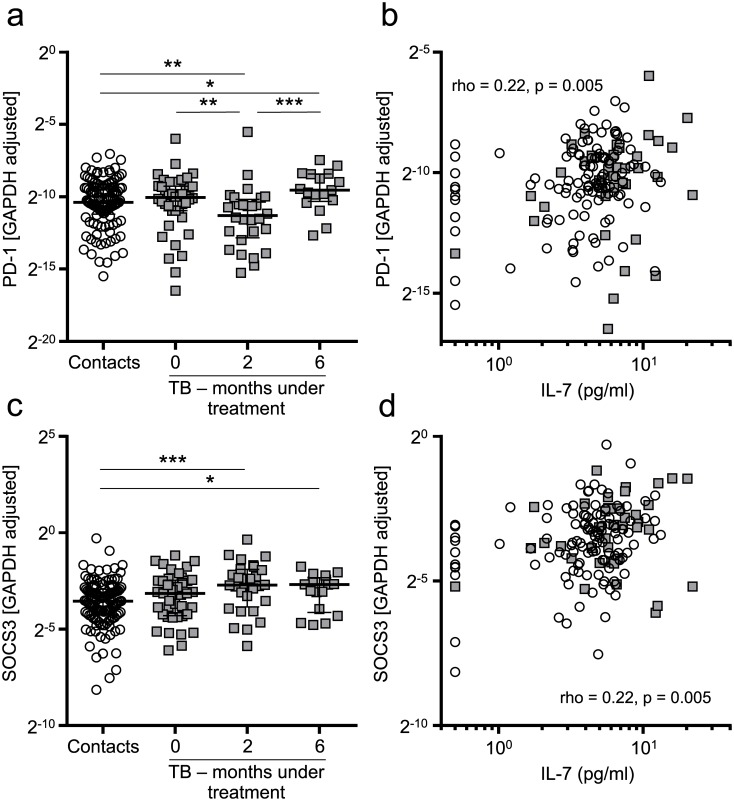
PD-1 and SOCS3 mRNA expression of CD4+ T cells from tuberculosis patients and healthy contacts. The expression of PD-1 **(a)** and SOCS3 **(c)** was determined for mRNA isolated from CD4^+^ T cells, using Glyceraldehyde 3-phosphate dehydrogenase (GAPDH) as a reference. Cycle threshold differences (2^-ΔCt^) are shown for healthy contacts [n = 117 (PD-1), n = 119 (SOCS3)], and for tuberculosis patients prior to (0 months, n = 40), during (2 months, n = 28), and after (6 months, n = 17) treatment. Median and interquartile range is depicted. Spearman correlation between plasma IL-7 and **(b)** PD-1 or **(d)** SOCS3 for healthy contacts (circles) or tuberculosis patients (squares) prior to treatment. Each symbol indicates mean values of duplicates from each individual donor. Due to a low overlap between tuberculosis patients, exact Mann-Whitney U test used for comparison of all groups. Nominal p-values are indicated as * p < 0.05, ** p < 0.01, *** p < 0.001.

Programmed cell death (PD)-1, a marker of T-cell exhaustion and senescence was recently found to be expressed on T cells with impaired response to IL-7 [14]. We determined PD-1 mRNA expression of purified CD4^+^ T cells and found similar PD-1 expression among healthy contacts and tuberculosis patients prior to therapy (Fig 6a). Under therapy, a decrease of PD-1 expression was found for tuberculosis patients (p = 0.007) followed by an increase until recovery (p < 0.001). PD-1 levels in recovered tuberculosis patients were even higher as compared to healthy contacts (p = 0.04). We found a moderate but significant positive correlation of PD-1 (rho = 0.22, p = 0.005) with IL-7 (Fig 6b). Previously, we identified SOCS3 as a marker of CD4^+^ T cells in tuberculosis [48], and others described SOCS3 as a central regulator of T-cell exhaustion and target of IL-7 in chronic viral infections [10]. Therefore we determined SOCS3 mRNA expression of CD4^+^ T cells. Marginal increased SOCS3 expression was detected in tuberculosis patients prior to therapy (p < 0.16), and significantly increased SOCS3 levels were detected at two months under therapy (p < 0.001) and after six months (p = 0.04) as compared to healthy contacts (Fig 6c). As for PD-1, a moderate positive correlation between SOCS3 expression and IL-7 concentrations was found (rho = 0.22, p = 0.005) (Fig 6d). We concluded that expression of T-cell exhaustion marker SOCS3 was increased in tuberculosis patients during therapy but was only moderately associated with aberrant IL-7 plasma concentrations. These observations indicated similarities and differences of aberrant IL-7 pathway features in tuberculosis patients as compared to AIDS patients.

## Discussion

In the presented study, we identified alterations in the IL-7 pathway and impaired T-cell response to IL-7 co-stimulation in tuberculosis patients.

First, we detected higher IL-7 plasma concentrations in tuberculosis patients that decreased during therapy and recovery. Lymphopenia may cause high IL-7 plasma levels [49, 50] and few reports indicated a role of lymphopenia in tuberculosis [51–53], but this has not been verified by others [54]. We did not determine lymphocyte counts in the present study and cannot prove or refute lymphopenia as a possible cause for high IL-7 levels. However, there is evidence that IL-7 serum concentrations are affected only at very low CD4^+^ T-cell numbers in AIDS patients [41, 55], and these levels are far below lymphopenia described in tuberculosis [49, 50]. Another possible explanation for higher IL-7 plasma concentrations is impaired receptivity/consumption of IL-7 by T cells [21]. Our investigations provide evidence for reduced mIL-7R expression and impaired IL-7 co-stimulatory effects on T cells from tuberculosis patients. Strong evidence for impaired IL-7 regulation and T-cell function was found for chronic viral infections, especially AIDS [56]. In AIDS patients increased IL-7 plasma levels and decreased mIL-7R expression of T cells were described [17, 42, 43, 57–59]. Furthermore, impaired T-cell response to IL-7 in AIDS patients was shown to affect immune reconstitution during anti-retroviral therapy [13, 60, 61]. In order to determine possible dependencies between mIL-7R expression on T cells and IL-7/sIL-7R plasma concentrations, we performed correlation analyses. For contacts there was a tendency of positive correlation between mIL-7R and sIL-7 levels, whereas IL-7 plasma levels showed a marginal negative correlation with mIL-7R expression. Given the described regulatory influence of IL-7/sIL-7R on mIL-7R expression [21], we speculate that IL-7 and sIL-7R plasma level alterations caused by tuberculosis disrupted this dependency that indicates the homeostatic balance in healthy individuals. The low number of samples included for mIL-7R analyses restricted the validity of these results. In addition, analyses of mIL-7R during disease course and after recovery are needed to confirm this thesis.

Several mechanisms and T-cell phenotypes were described to play a role in impaired IL-7 functions of AIDS patients. Chronic inflammation and increased serum concentrations of IL-6 were found in HIV/AIDS [14, 15], and functional *in vitro* assays indicated inhibitory effects of the pro-inflammatory cytokines IL-6 and IL-1β on IL-7-mediated signal transduction [15]. Higher IL-6 plasma concentrations were described for tuberculosis [47, 62], and we confirmed higher IL-6 plasma concentrations in a subgroup of tuberculosis patients in the present study. However, we did not detect IL-7 response differences between IL-6_high_ and IL-6_low_ subgroups among tuberculosis patients (Fig 5e and 5f). Hence there was no indication for an association between IL-6 plasma concentrations and impaired IL-7 T-cell response of tuberculosis patients.

T-cell exhaustion was found in AIDS patients [12, 13, 60, 63–65] and was associated with decreased IL-7R expression [60, 64] and impaired IL-7 response [13]. Initial studies indicated a role of T-cell exhaustion in tuberculosis animal models [7, 8]. Our results on SOCS3 and PD-1 expression did not support a major role of T-cell exhaustion in human tuberculosis and this is in accordance with a previous study [66]. These differences might at least partly be due to the fact that exhaustion is poorly defined for CD4^+^ T cells in contrast to CD8^+^ T cells [67]. Therefore, other marker molecules may be indicative for exhaustion in CD4^+^ T cells. We were not able to study the phenotype of CD8^+^ T cells in detail in the present study but decreased mIL-7R expression (Fig 3a) may indicate exhaustion of CD8^+^ T cells in tuberculosis patients.

Impaired mIL-7R signalling was described for T cells from AIDS patients [13, 14, 18, 68]. We detected lower STAT5 phosphorylation and showed also impaired IL-7 promoted cytokine release in T cells from tuberculosis patients. The capacity of IL-7 to promote IFNγ-expressing T cells for detection of *M*. *tuberculosis* infection has been shown before [46]. Here we provide first evidence that IL-7 mediated increased sensitivity of T cells to stimulation (e.g. by decreasing the T-cell receptor activation threshold [69]) was impaired in tuberculosis patients. One may therefore speculate that impaired IL-7 response not only hampered generation of effective memory but also effector T-cell response against acute tuberculosis. This raised the question if impaired T-cell response to IL-7 can be interpreted as a feature of T-cell anergy. Anergy is defined as unresponsiveness of T cells to their cognate antigen and anergy against PPD—measured by tuberculin skin test—has been described for tuberculosis patients before [70]. We did not detect differences in the PPD response of CD4^+^ T cells between tuberculosis patients and healthy contacts in the present study (Fig 5b). However, we would speculate that impaired T-cell responses to IL-7 contributed to the phenomenon of diminished tuberculin reactivity in tuberculosis patients as this *in vivo* test would be better reflected by IL-7-supplemented PPD stimulation in our *in vitro* assay. Since IL-7 effects on T-cell function include a decreased T-cell receptor activation threshold [69], impaired mIL-7R signaling may contribute to diminished T-cell receptor signaling characteristic for T-cell anergy [71]. Therefore impaired mIL-7R signaling may contribute to tuberculin skin test anergy described for tuberculosis patients but additional studies are needed to further clarify the exact role of IL-7.

We also detected lower sIL-7R plasma concentrations in tuberculosis patients and normalisation during therapy and recovery. sIL-7R levels were previously shown to affect IL-7-availability for T cells, but the role of aberrant sIL-7R levels in immune pathologies is a matter of controversy [26–28]. Crawley et al. detected increased sIL-7R concentrations in plasma samples from AIDS patients and described sIL-7R-Fc chimera-mediated inhibition of IL-7 bioactivity [28]. They hypothesised that increased sIL-7R concentrations limited availability of IL-7 for T cells [28]. In contrast, Rose et al. found decreased sIL-7R plasma concentrations in HIV/AIDS patients as compared to controls [31]. sIL-7R plasma concentrations of this study were similar to the present study and 5 to 10 times lower for both study groups as compared to the study published by Crawley et al. [28]. Recently, Lundstrom et al. proposed an alternative model of IL-7 storage provided by the sIL-7R [26]. They demonstrated that sIL-7R even potentiates the bioactivity of IL-7 by forming a reservoir of accessible IL-7 [26]. In accordance, high sIL-7R as well as IL-7 plasma concentrations were associated with multiple sclerosis, and sIL-7R had potentiating effects on exacerbation of experimental autoimmune encephalomyelitis [26]. From this, they concluded that increased plasma concentrations of sIL-7R supported generation of autoimmunity by promoting IL-7-dependent T cells [26]. Since IL-7 serum levels are predominantly regulated by T-cell consumption [21], both restriction and reservoir hypotheses suggest dependency of IL-7 on sIL-7R levels. In the present study, we did not detect a correlation between IL-7 and sIL-7R plasma levels in tuberculosis patients or healthy contacts, although both factors were affected during tuberculosis pathogenesis. It is therefore tempting to speculate that sIL-7R has either no regulatory activities on IL-7, or that additional factors influence sIL-7R and/or IL-7 serum levels. In accordance, the proposed regulatory function of sIL-7R on IL-7 has been questioned by others [72].

We evaluated the utility of IL-7 and sIL-7R plasma concentrations as biomarkers for diagnosis of active tuberculosis using ROC curve and Random Forest-based statistics. Both markers showed moderate classification capacity and the combined efficacy of both markers revealed correct prediction for 73% of all donors. Since normalization of low sIL-7R and high IL-7 plasma concentrations during recovery from tuberculosis was found, these parameters may qualify as biomarker candidates for successful tuberculosis chemotherapy. This study was not designed to evaluate markers for the efficacy of tuberculosis therapy but future studies may address this important question.

Immunomodulatory therapies of tuberculosis gained increasing interest during recent years to complement antibiotic therapy that is periled e.g. by multi-drug resistant mycobacteria [73]. IL-7 is a promising candidate for immunotherapies and is already applied in clinical trials against chronic viral infections [74, 75]. However, the mechanisms underlying impaired IL-7 signalling pathways during chronic infections may antagonise IL-7-based novel therapy strategies. Our study contributed to the characterisation of impaired IL-7 T-cell response that may indeed counteract IL-7 treatment in tuberculosis.

We provide initial evidence that IL-7-availability is not critical during tuberculosis. Instead, T-cell functions in response to IL-7 are impaired, and therefore approaches targeting T-cell abnormalities—causative for reduced IL-7 response—may be helpful. Since IL-7 availability is a crucial factor for the development of memory T-cell induction [76], such an approach might also aim at improving protection against recurrent *M*. *tuberculosis* infection and disease.

## Methods

### Study design and samples

In this hospital-based observational study, we recruited adult tuberculosis patients (n = 57; Table 1) and exposed but healthy household contacts (healthy contacts) (n = 151). Tuberculosis patients were recruited at the Komfo Anokye Teaching Hospital (KATH), the Kumasi South Hospital (KSH), and the Kwame Nkrumah University of Science and Technology (KNUST) Hospital, Ghana, in 2011–2012. Diagnosis of tuberculosis was based on patient history, chest X-ray, and sputum smear test. For sputum smear negative cases, laboratory confirmation by *M*. *tuberculosis* sputum culture was performed. Tuberculosis patients with a known history of HIV infection were excluded from this study. Chemotherapy according to the Ghanaian guidelines was initiated immediately after the first blood sample was taken. For the patient study group, peripheral heparinised blood was taken consecutively (i.e. prior to treatment, under treatment (at 2 months), and after recovery (at 6 months)). Only a subgroup of tuberculosis patients (n = 36) completed the study procedure. Twenty-one tuberculosis patients were not included at all time points, including nine patients included only prior to treatment; six patients prior to treatment and under treatment; two patients prior to treatment and after recovery, and four patients during treatment and after recovery. Healthy tuberculosis patient contacts (short: healthy contacts) were recruited at the homes of tuberculosis index cases and showed no clinical symptoms of tuberculosis. A subgroup of healthy contacts (n = 19) and tuberculosis patients (n = 32) was tested for *M*. *tuberculosis* PPD-specific immune response before and showed significant IFNγ expression [77]. We took heparinised blood (up to 30 ml) from each donor. Not all samples were included for all experiments, and the respective numbers of samples included are given in the figure legends. A second cohort of tuberculosis patients (n = 22) and healthy contacts (n = 24) were recruited in the period of October 2015 to March 2016. HIV-positive individuals were excluded from the analysis (First Response HIV 1–2.0 Card Test, Premier Medical Corporation).

### Ethics statement

All study participants were adults who gave written informed consent. All participants were free to drop out at any time of the study. The studies were approved by the Committee on Human Research, Publication and Ethics (CHRPE) at the School of Medical Sciences (SMS) at the Kwame Nkrumah University of Science and technology (KNUST) in Kumasi, Ghana.

### Measurement of sIL-7R concentrations using cytometric bead assay

Peripheral blood mononuclear cells (PBMCs) were isolated from heparinised whole blood (diluted 1:1 in PBS) by density centrifugation (Ficoll, Biochrom) according to manufacturer’s instructions. PBMCs were cryopreserved in DMSO/FCS (each 10%) containing medium. The plasma layer (diluted 1:1 in PBS) were collected and frozen at -80°C until processing. Diluted plasma samples were thawed in parallel and analysed for sIL-7R expression. Quantification of sIL-7R was performed according to the protocol of Faucher et al. [78] with minor modifications. In brief, we applied cytometric bead array (CBA) (Bead A4, BD Biosciences). Conjugation of beads with polyclonal goat anti-human CD127 (IL-7Rα) antibody (R&D Systems, AF306) was done according to manufacturer’s instructions. Biotinylated mouse anti-human CD127 (clone HIL-7R-M21, BD Biosciences) was used as detection antibody. Samples were incubated with labelled beads in PBS for 1 hour at room temperature and then the detection antibody (5 μl) was added for overnight incubation in the fridge. Afterwards, Streptavidin-PE (1 μl) (Southern Biotech) was added and incubated for 30 min at room temperature. Finally the beads were washed twice in PBS. For analyses, the bead pellets were resuspended in 80 μl PBS and analysed using a BD LSRFortessa flow cytometer (BD Biosciences) and the FCS Express 4 (De Novo Software) software. For absolute quantification, the assay was calibrated with dilutions of rhIL-7R alpha-Fc chimera (R&D Systems). sIL-7R concentrations were calculated using the non-linear regression tool of GraphPad Prism 6 (Graphpad Software Inc.). Possible effects of IL-7 on sIL-7R measure were excluded by Faucher et al. [78].

### Measurement of plasma IL-6 and IL-7

IL-6 and IL-7 was determined in duplicate for diluted plasma samples using Human IL-6 ELISA Ready-SET-Go! (eBioscience) and Human IL-7 Quantikine HS ELISA kit (R&D Systems), respectively, according to manufacturer’s instructions. Samples were measured using the Infinite M200 ELISA reader (Tecan). Concentrations were calculated from the respective standard curves by applying 4-parametric logistic regression. Samples outside the detection range were set to the corresponding lower or upper range value.

### Real-Time PCR of IL-7R variants and T-cell exhaustion markers

CD4^+^ cells were isolated from freshly isolated PBMCs (1.5 x 10^7^ cells) using anti-human CD4 magnetic particles (BD Biosciences) according to manufacturer’s recommendations. Cell purity was evaluated by flow cytometry and was generally higher than 95%. miRNA was isolated from at least 5 x 10^6^ enriched CD4^+^ cells using mirVanaTM miRNA Isolation Kit (Life Technologies) following manufacturer’s instructions. cDNA was generated by Maxima H Minus First Strand cDNA Synthesis kit (Thermo Scientific), while RT-PCR was performed with the QuantiTect SYBR Green PCR kit (Qiagen) for full-length IL-7R (H20: forward 5’-AATAATAGCTCAGGGGAGATGG-3’, reverse 5’-ATGACCAACAGAGCGACAGAG-3’), IL-7R lacking exon 6 (H6: forward 5’-GATCAATAATAGCTCAGGATTAAGC-3’, reverse 5’-AAGATGTTCCAGAGTCTTCTTATG-3’), and IL-7R lacking exon 5–6 (H5-6: forward 5’-ATGAAAACAAATGGACGGATTAAGC-3’, reverse 5’-AAGATGTTCCAGAGTCTTCTTATG-3’), PD-1 (forward 5’-CTCAGGGTGACAGAGAGAAG-3’, reverse 5’-GACACCAACCACCAGGGTTT-3’), SOCS3 (forward 5’-GACCAGCGCCACTTCTTCAC-3’, reverse 5’-CTGGATGCGCAGGTTCTTG-3’) using glyceraldehyde 3-phosphate dehydrogenase (GAPDH) as housekeeping control gene (forward 5’-CACCATCTTCCAGGAGCGAG-3’, reverse 5’-GACTCCACGACGTACTCAGC-3’). The reaction with a final volume of 25 μl was run 2 min. at 50°C, 10 min. at 95°C, 45 cycles of 15 s at 95°C, 30 s at 53°C and 30 s at 72°C, followed by a melt curve sequence of 15 s at 95°C, 60 s at 60°C with a slow gradient to 95°C and finally 15 s at 60°C. Data from duplicate reactions was evaluated using the 2^-ΔCt^ method. A 7500 Real-Time PCR machine (Applied Biosystems) was used for quantitative PCR analyses.

### Genotyping of IL7RA single nucleotide polymorphism

DNA was isolated from PBMCs using QIAamp DNA Mini Mini kit (Qiagen) followed by rs6897932C>T genotyping using a predesigned TaqMan SNP Genotyping Assay (Applied Biosystems) following manufacturer’s instructions.

### Staining of PBMCs

Frozen PBMCs were thawed and washed with RPMI 1640 supplemented with 10% foetal calf serum (FCS), 2 mM L-Glutamine, 10 mM HEPES, and 50 U/ml Penicillin-Streptomycin (all from Thermo Fisher). Cells were stained with Viability Dye eFluor 780 (eBioscience) and antibodies against CD3 (PE-labelled, clone HIT3a, BD Biosciences), CD4 (BrilliantViolet510-labelled, clone OKT4, BioLegend), CD8 (PerCP-Cy5.5-labelled, clone HIT8a, BioLegend), CD25 (PE-Cy7-labelled, clone 2A3, BD Biosciences) and CD127 (AlexaFluor647-labelled, clone HIL-7R-M21, BD Biosciences). After cell wash, PBMCs were fixed with Fixation Buffer (BioLegend) and subsequently analysed using a BD LSRFortessa flow cytometer (BD Biosciences). Gating procedures are depicted in S3 Fig. For detection of mIL-7R in the second independent cohort of tuberculosis patients and healthy contacts we used the CD127 antibody clone A019D5 (BioLegend). Comparison of both antibody clones revealed similar T-cell binding pattern as well as percentages of mIL-7R_high_ and mIL-7R_low_ T cells.

### STAT5 phosphorylation by IL-7

Freshly isolated PBMCs were stained for CD4 (AlexaFluor488, clone RPTA-4, BioLegend) followed by addition of 100 μl pre-warmed X-VIVO 15 medium (Lonza) added 50 U/ml Penicillin-Streptomycin with or without human recombinant IL-7. The concentration of IL-7 was titrated prior to the study and a concentration of 10 ng/ml was sufficient to induce pSTAT5 in 94% of the T cells (S5 Fig). Higher IL-7 concentrations (25 or 50 ng/ml) did not further increase STAT5 phosphorylation (S5 Fig). Therefore we cultured the samples with and w/o 10 ng/ml of recombinant human IL-7 in this study. After 15 min incubation at 37°C, 5% CO_2_, cells were fixed for 15 min. with 100 μl 1x True-Nuclear Transcription Factor buffer (BioLegend). Subsequently, cells were permeabilised with 100% methanol, washed in PBS/10% FCS and stained for p-STAT5 Y694 (PE, clone SRBCZX, eBioscience). Analysis was performed on a BD Accuri C6 flow cytometer. Gating procedure is shown in S5 Fig.

### *Ex vivo* stimulation of whole blood

Heparinised blood was diluted 1:2 in RPMI 1640 supplemented with 2 mM L-Glutamine and 50 U/ml Penicillin-Streptomycin in a 96-well U bottom plate. Cells were stimulated with 10 μg/ml PPD (Statens Serum Institute) and/or 10 ng/ml recombinant human IL-7 (BioLegend), or left unstimulated. After 2.5 hours of stimulation at 37°C, 5% CO_2_, Brefeldin A (Sigma Aldrich) was added at a concentration of 3.75 μg/ml followed by 16 hours of incubation. Erythrocytes were subsequently lysed in two rounds by resuspending pelleted cells in 100 μl RBC Lysis Buffer (Roche) followed by 10 min incubation at room temperature. Next, cells were fixed and permabilised (BioLegend) and stained with antibody against CD4 (AlexaFluor488, clone RPTA-4, BioLegend), IFNγ (PE, clone 25723.11, BD Biosciences) and CD154 (APC, clone 24.31, BioLegend). Cells were analysed using a BD Accuri C6 flow cytometer (BD Biosciences). Gating procedure is shown S6 Fig.

### Statistical analysis

Statistical analyses were performed using R version 3.3.0, applying Exact Mann-Whitney U test from the package *coin* for comparison between groups and Wilcoxon signed-rank test for evaluation of repeated measurements. Spearman correlation was used to evaluate association between continuous variables, while Receiver Operating Characteristic (ROC) was performed using the package *ROCR*. Random forest analysis was performed with the package *ranger*, applying 10^5^ random trees and adjusting the importance measure by permutation. Plots were generated in R and GraphPad Prism version 6.07.

## Supporting information

S1 Table Correlation between mIL-7R expression on T cells and plasma IL-7 or sIL-7R concentrations. (EPS)

S1 Fig Gender differences in plasma sIL-7R levels. Plasma concentrations of sIL-7R from TB contacts (n = 149) and tuberculosis patients prior to (0 months, n = 52), during (2 months, n = 46) and after (6 months, n = 41) treatment was determined by cytometric bead array. Exact Mann-Whitney U test used for comparison of gender differences.
(PDF)

S2 Fig Changes in plasma sIL-7R and plasma IL-7 during chemotherapy. Absolute (left panel) and relative (right panel) differences of a) plasma sIL-7R and b) plasma IL-7 level after (6 months) and prior treatment for tuberculosis. Concentration of sIL-7R in plasma from TB patients was determined by cytometric bead array, while plasma IL-7 level was determined by ELISA (n = 36). p-values for Spearman correlation are shown, while linear regression lines are shown for guidance.
(PDF)

S3 Fig Gating strategy for IL-7R_low_, and IL-7R MFI of CD4^+^ and CD8^+^ cells. Proportions (%) of cells within the individual gates are indicated.
(PDF)

S4 Fig Surface level of IL-7R on CD3^+^CD4^+^ and CD3^+^CD4^-^ cells. Heparinised blood from TB patients (n = 22) and contacts to TB patients (n = 24) was lysed (RBC Lysis Buffer, Roche) and leukocytes were stained for CD3 APC (clone UCHT1, BD Biosciences), CD4 AlexaFluor 488 (clone RPTA-4, Biolegend) and IL-7R (CD127) PE-Cy7 (clone A019D5, Biolegend). Cells were analysed on a BD Accuri C6 Flow Cytometer (BD Biosciences). Mean Fluoresence Intensity (MFI) of IL-7R is shown for (a) CD3^+^CD4^+^ and (b) CD3^+^CD4^-^ cells. Exact Mann-Whitney U test is used for comparison of groups.
(PDF)

S5 Fig STAT5 phosphorylation of CD4^+^ cells after IL-7 stimulation. (a) Gating strategy for STAT5 phosphorylation (pSTAT5) on CD4^+^ cells stimulated with (solid line) or without (shaded) 10 ng/ml IL-7 for 15 min. Proportions (%) of cells in the individual gates are indicated, and mean fluorescence intensity (MFI) is shown for the two stimulations. (b) Titration of IL-7. PBMCs stimulated as in (a) with various concentrations of IL-7 shown for CD4^+^ cells.
(PDF)

S6 Fig Gating strategy for IFNγ^+^CD40L^+^ cells. Gating strategy for IFNγ^+^CD40L^+^ cells of CD4^+^ cells after overnight stimulation of whole blood with PPD. Proportions (%) of cells in the individual gates are indicated.
(PDF)
