# Supplementary figures and images for: Aberrant plasma IL-7 and soluble IL-7 receptor levels indicate impaired T-cell response to IL-7 in human tuberculosis
Source: PLoS Pathog. 2017 Jun 5;13(6):e1006425. doi: 10.1371/journal.ppat.1006425 (PMC5472333; doi:10.1371/journal.ppat.1006425)

**S1 Fig**

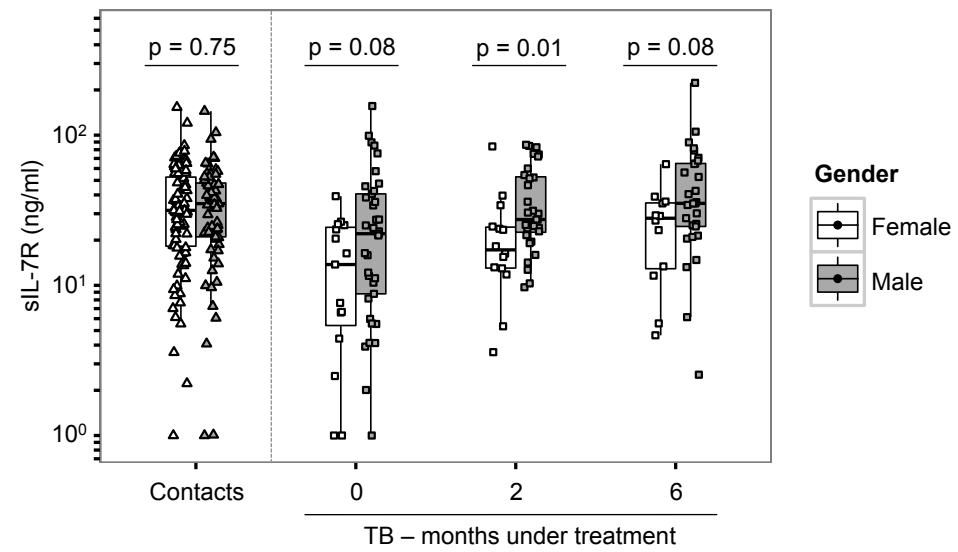

Supplement: S1 Fig — Plasma concentrations of sIL-7R from TB contacts (n = 149) and tuberculosis patients prior to (0 months, n = 52), during (2 months, n = 46) and after (6 months, n = 41) treatment was determined by cytometric bead array. Exact Mann-Whitney U test used for comparison of gender differences. (PDF) [file ppat.1006425.s002.pdf]

**S2 Fig**

**a**

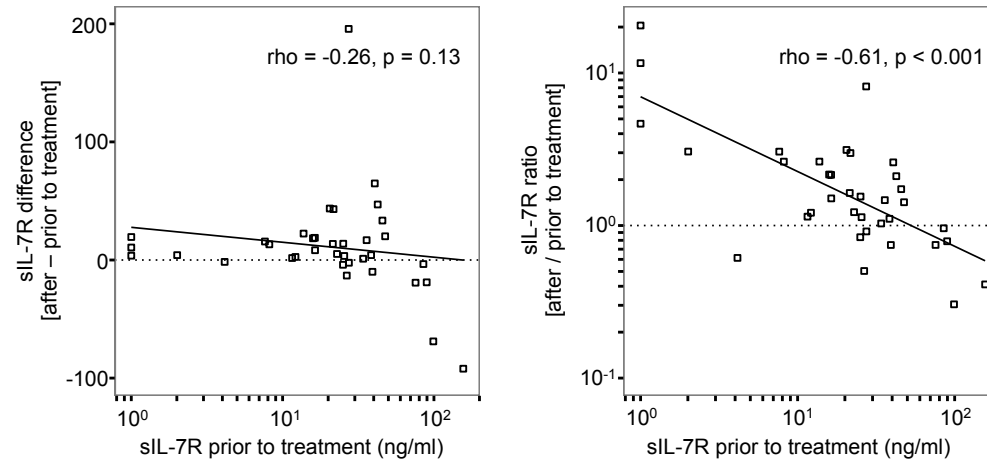

**b**

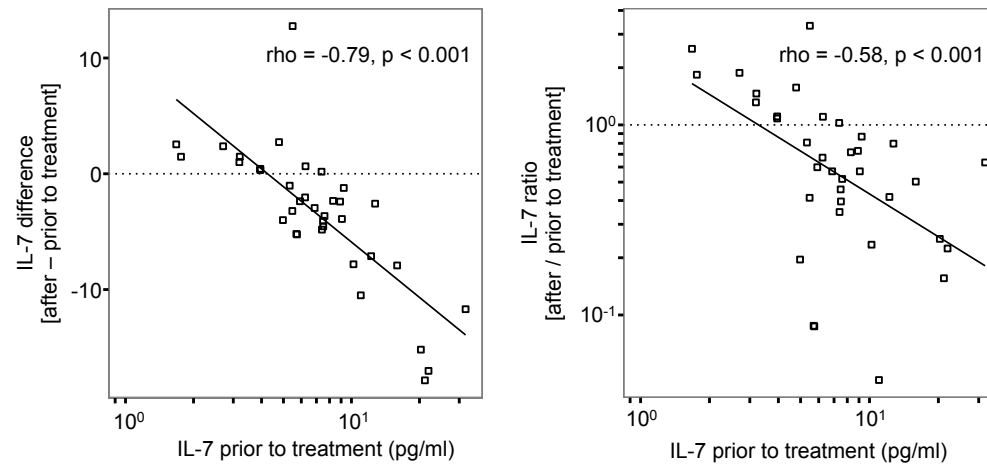

Supplement: S2 Fig — Absolute (left panel) and relative (right panel) differences of a) plasma sIL-7R and b) plasma IL-7 level after (6 months) and prior treatment for tuberculosis. Concentration of sIL-7R in plasma from TB patients was determined by cytometric bead array, while plasma IL-7 level was determined by ELISA (n = 36). p-values for Spearman correlation are shown, while linear regression lines are shown for guidance. (PDF) [file ppat.1006425.s003.pdf]

S3 Figure

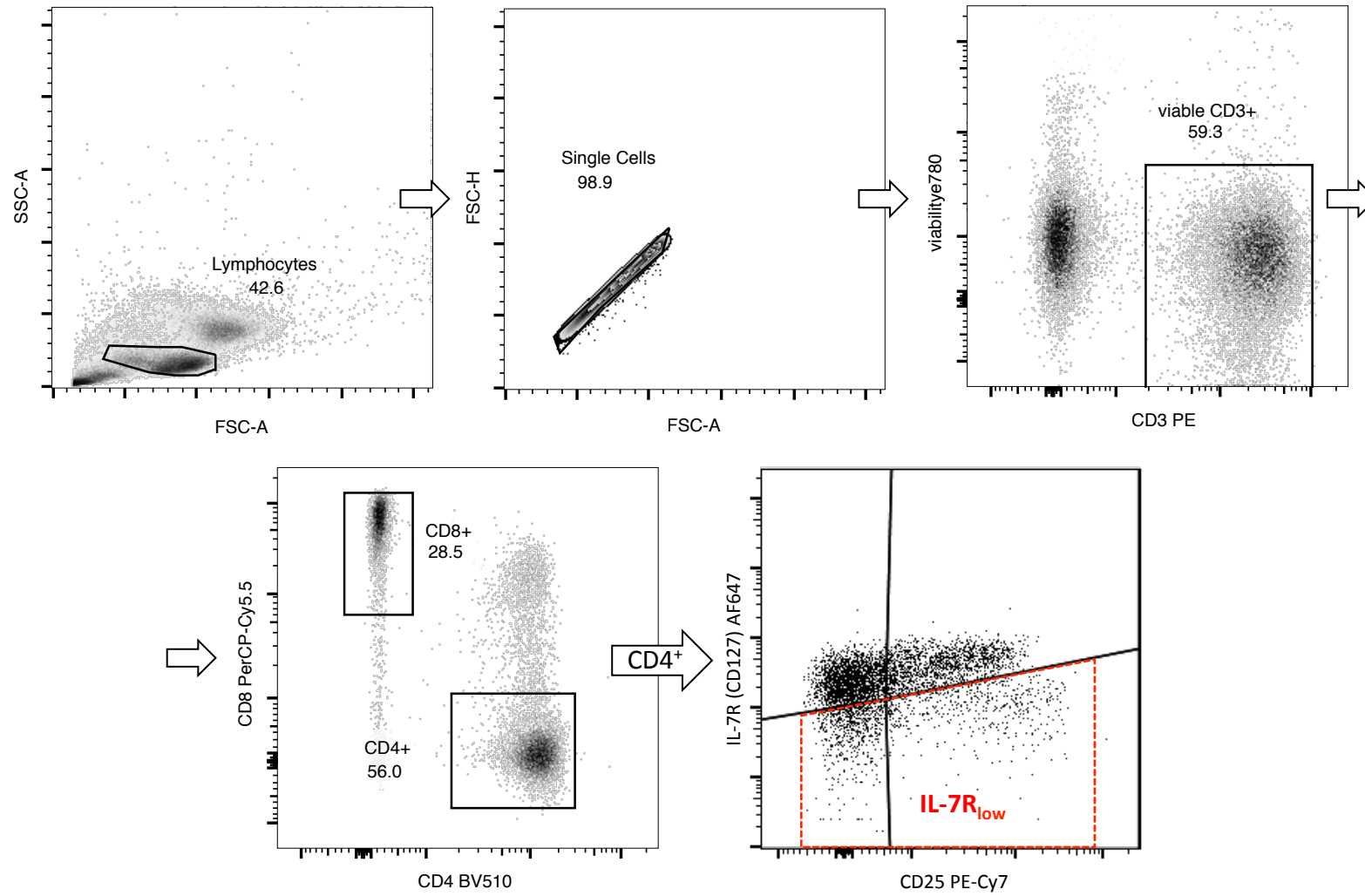

Supplement: S3 Fig — Proportions (%) of cells within the individual gates are indicated. (PDF) [file ppat.1006425.s004.pdf]

**S4 Fig**

**a**

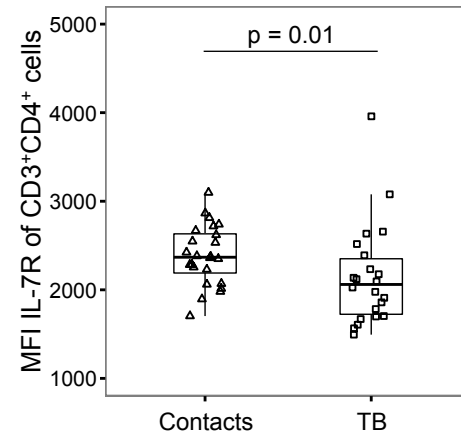

**b**

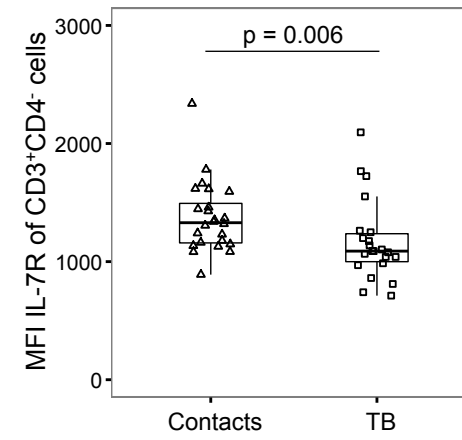

Supplement: S4 Fig — Heparinised blood from TB patients (n = 22) and contacts to TB patients (n = 24) was lysed (RBC Lysis Buffer, Roche) and leukocytes were stained for CD3 APC (clone UCHT1, BD Biosciences), CD4 AlexaFluor 488 (clone RPTA-4, Biolegend) and IL-7R (CD127) PE-Cy7 (clone A019D5, Biolegend). Cells were analysed on a BD Accuri C6 Flow Cytometer (BD Biosciences). Mean Fluoresence Intensity (MFI) of IL-7R is shown for (a) CD3+CD4+ and (b) CD3+CD4- cells. Exact Mann-Whitney U test is used for comparison of groups. (PDF) [file ppat.1006425.s005.pdf]

**S5 Fig**

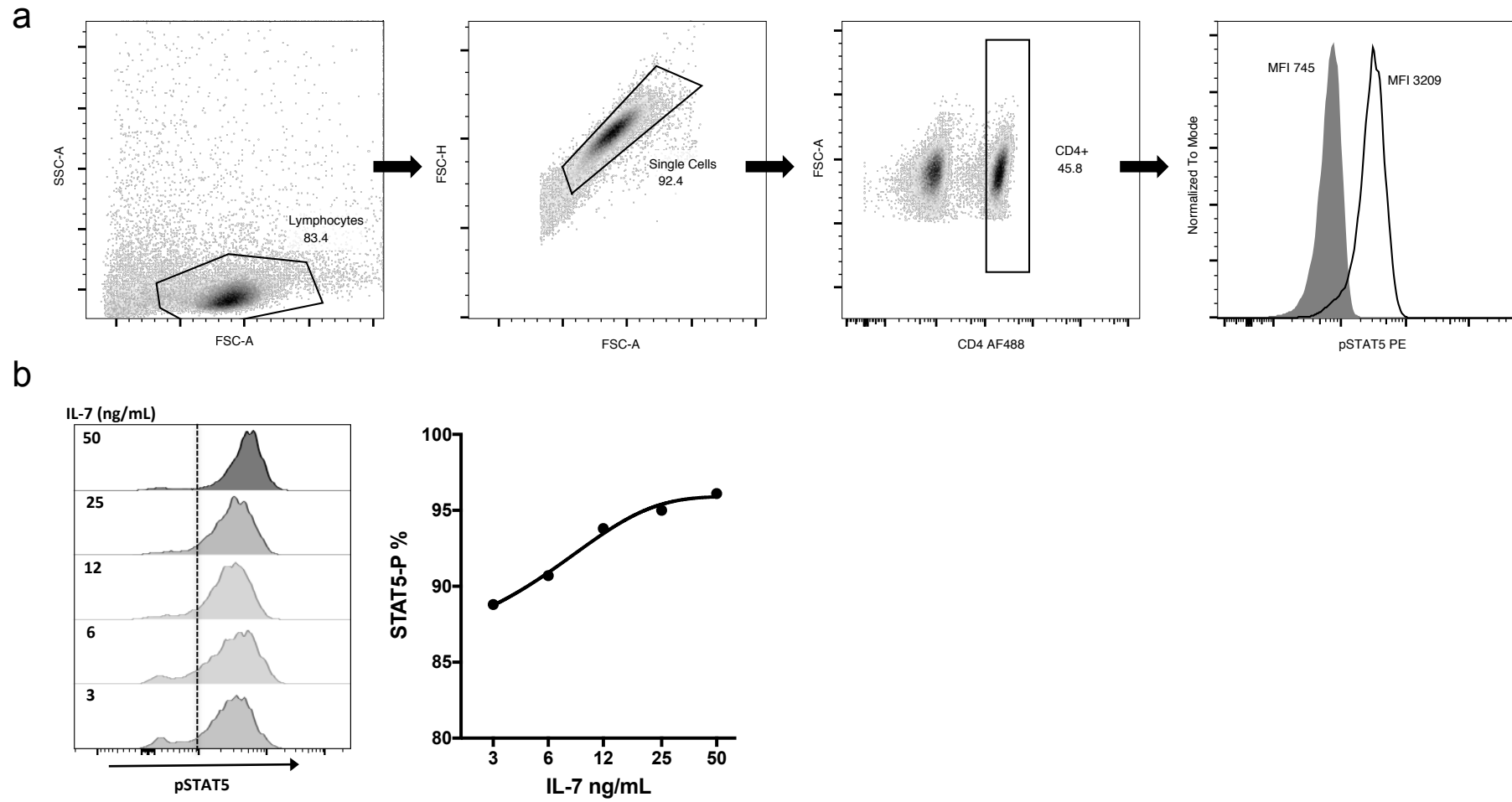

Supplement: S5 Fig — (a) Gating strategy for STAT5 phosphorylation (pSTAT5) on CD4+ cells stimulated with (solid line) or without (shaded) 10 ng/ml IL-7 for 15 min. Proportions (%) of cells in the individual gates are indicated, and mean fluorescence intensity (MFI) is shown for the two stimulations. (b) Titration of IL-7. PBMCs stimulated as in (a) with various concentrations of IL-7 shown for CD4+ cells. (PDF) [file ppat.1006425.s006.pdf]

**S6 Fig**

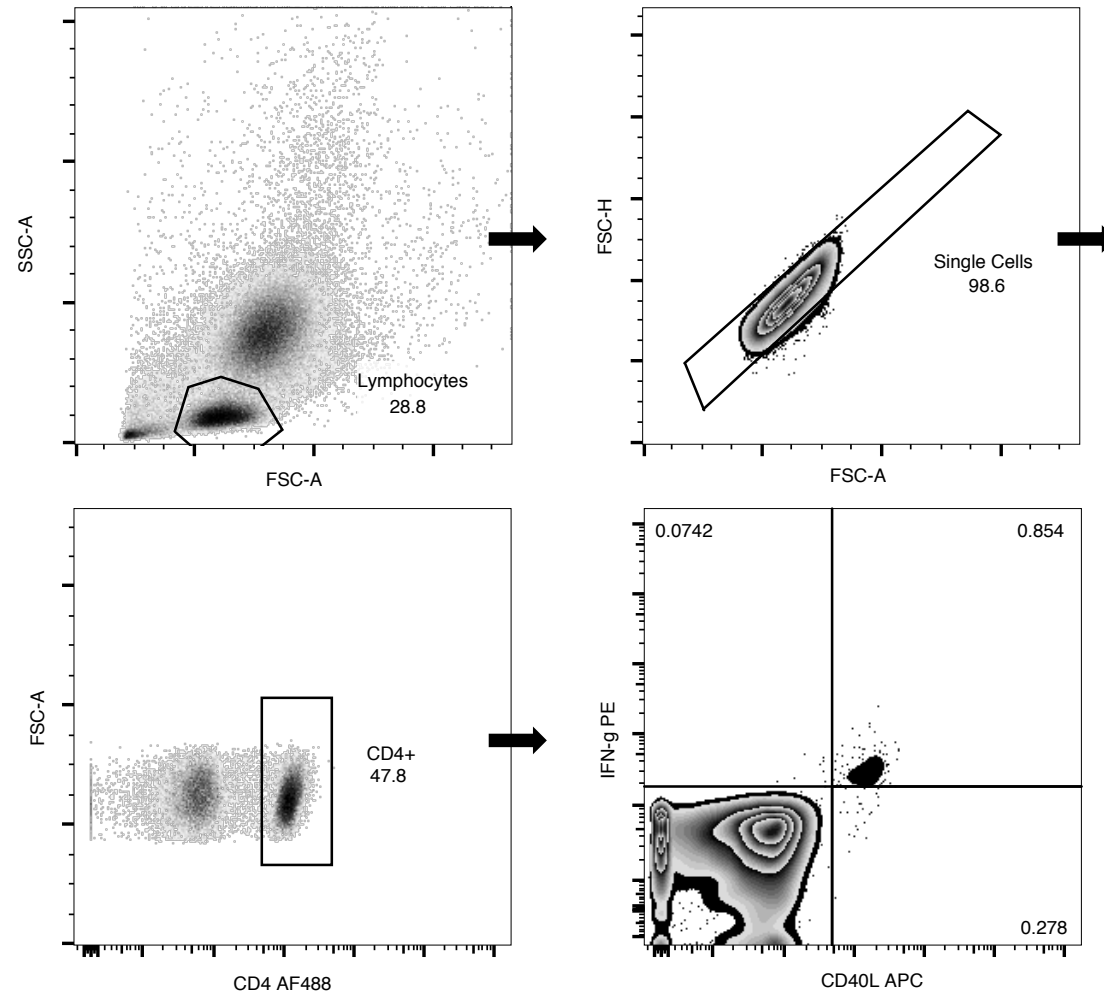

Supplement: S6 Fig — Gating strategy for IFNγ+CD40L+ cells of CD4+ cells after overnight stimulation of whole blood with PPD. Proportions (%) of cells in the individual gates are indicated. (PDF) [file ppat.1006425.s007.pdf]
